# Lower pole calculi larger than one centimeter: Retrograde intrarenal surgery

**DOI:** 10.4103/0970-1591.44266

**Published:** 2008

**Authors:** Andreas J. Gross, Thorsten Bach

**Affiliations:** Department of Urology, Asklepios Hospital Barmbek, Hamburg, Germany

**Keywords:** Lower pole calculi, percutaneous nephrolithotomy, ureterorenoscopy, urolithiasis

## Abstract

Controversy remains on how to treat lower pole calculi between 1 and 2 cm of size. Treatment options like shock wave lithotripsy (SWL) or percutaneous stone treatment (PCNL) are associated with poor stone-free rates or high morbidity.

Due to the ongoing development in endourologic technology, especially in flexible renoscopy, laser technique and grasping devices (tipless Nitinol baskets) retrograde intrarenal surgery (RIRS) has become an option in treating these patients.

Based on personal experience and an overview of the published literature we discuss RIRS as a valuable alternative to PCNL in treating patients with larger calculi of the lower pole.

The technical developments in laser technology as well as significant improvement in flexible renoscopes have made RIRS for larger lower pole stones possible. The low complication rate gives RIRS for lower pole stones superiority over the invasive percutaneous approach, which is associated with significant morbidity, even in experienced hands.

## INTRODUCTION

When counseling the guidelines of the numerous urological societies different treatment options for lower pole calculi are recommended. For lower pole calculi smaller than 1 cm, shock wave lithotripsy (SWL) is recommended as first-choice treatment. On the other hand, percutaneous nephrolithotomy (PCNL) is accepted as first-line treatment for larger and complex kidney stones,[1] especially in staghorn calculi.

However, there is controversy on how to treat lower pole calculi between 1 and 2 cm of size. Of course SWL remains a treatment option, but stone-free rates are poor and retreatment rates are high.[2,3] In many patients with larger stones the percutaneous approach seems to be appropriate, but although stone clearance is high with this approach,[4] it remains an invasive procedure with relevant morbidity.[5]

With ongoing development in not only laser devices and fiber technique but even more in handling and durability of semi-rigid and flexible renoscopes as well as in imaging techniques (chip-on-the-tip), retrograde intrarenal surgery (RIRS) turns out to be an option in patients with lower pole calculi between 1 and 2 cm of size.

Based on personal experience we report our own modus operandi of handling lower pole calculi treated in our institution. Furthermore, a Medline research, with special interest on stone clearance and complication rate was carried out reviewing the literature between 1984 and 2007 to give an overview of the literature.

## TECHNIQUE OF RETROGRADE INTRARENAL SURGERY

### Preparation of patients

Like in other procedures, careful patient selection and preparation is of paramount importance. All patients have to undergo a systematic diagnostic workup including stone size, evaluated radiographically (kidney-ureters-bladder (KUB) or abdominal computer tomography (ACT)) and by ultrasound. Furthermore the evaluation of the anatomical situation of the collecting system (intravenous pyelogram, retrograde pyelogram, ACT). Urinary tract infections are ruled out or treated preoperatively according to the antibiogram. Evaluation of serum creatinine and clotting parameter are determined. In our institution pre-renoscopy stenting is recommended (one week in advance), since it could be shown that stenting increases the possible in-toto stone extraction volume.[6]

Instruments and technique of retrograde intrarenal surgery for lower pole calculi

The procedure is performed with the patient positioned on a fluoroscopic table (Siemens Uro-Access), as described by Grasso and Ficazzola.[7] Patients are under general or spinal anesthesia.

### Intraoperative setup

At the beginning of the procedure the ureteral stent is removed and a retrograde pyelogram is carried out. Afterwards a safety wire (Terumo) is placed in the upper pole of the collecting system. This guide wire is optional, but in our institution highly recommended, if the workup of larger calculi is planned. A ureteral access sheath is placed, if we expect more than three passages through the urethra and the orifice. The use of a second guide wire allows the work with a safety wire after placing the access sheath. Furthermore, repeat placement of the access sheath during stone workup and extraction of the fragments becomes easier and faster, once a second guide wire is used.

### Stone workup

To access the lower pole, flexible ureterorenoscopes have proven their usefulness. Depending on the anatomy of the collecting system a flexible 6/8.8 Charriere (Charr.) (Wolf Viper) or a flexible 6.75/8.6 Charr. (ACMI Dur-8 Elite) renoscope, with active secondary deflection is used.

### At this point several options occur

First of all, the stone can be grasped with a basket and extracted in-toto, which is basically an option for smaller stones. Very small stones can be extracted through the access sheath. Another option is to work up the stone *in situ*, using a Holmium:YAG laser (45 Watt Holmium:YAG (Sphinx, Lisa laser, Katlenburg, Germany). Afterwards stone extraction is carried out, using a zero-tip Nitinol basket. The third option is to grasp the stone and pull it into the ureter without using any force until a stop-point is reached. Now the flexible renoscope is extracted and a semi-rigid 6/9.8 Charr. renoscope (Olympus) is used for stone workup using the Holmium:YAG laser. Fragments are removed afterwards.

Last but not the least the stone can be grasped and relocated into the upper pole of the collecting system, where stone workup proceeds, again after changing to a semi-rigid ureteroscope. Afterwards fragments are extracted. Changing from flexible to semi-rigid ureteroscopy gives the surgeon better flow and visualization during stone workup.

### Conclusion of the procedure

After complete stone workup and extraction of the fragments a retrograde pyelogram is shot to rule out residual fragments and extravasation. The placement of a ureteral stent is optional; the decision is left to the surgeon.

In the current literature it is shown that the new generation of flexible renoscopes has increased the therapeutic efficacy. Especially in combination with the Holmium:YAG laser morbidity could be decreased and success rate increased.[8,9] The reported stone-free rate ranges up to 95% within two ureterorenoscopic treatments, even for stones > 2 cm.[8] Minor and major complications are reported in 1.5-12% of cases.[9–11]

However, according to the guidelines of several urological societies SWL is recommended as first-choice treatment for small renal calculi. PCNL is accepted as first-line treatment for larger and complex kidney stones, especially in staghorn calculi.[1]

Nevertheless, in regard of the technical developments in recent years, especially in laser technique as well as in durability and functionality of smaller diameter flexible renoscopes, there is controversy on how to treat patients with lower pole calculi between 1 cm and 2-3 cm in size.

Although associated with a low complication rate, SWL does not very well in patients with lower pole stones above 1 cm, where stone-free rates as low as 29%[2,3] are reported.

### Percutaneous nephrolithotomy

PCNL has been advocated by some institutions as first-choice treatment in patients with lower pole calculi above 1 cm. Greatest benefit of PCNL is the high stone-free rate, independent of stone size within a single procedure. Stone-free rates between 78% and 100%[12] are described. However, on an average, single-procedure stone-free rates of 90% seem realizable.[13]

On the other hand, PCNL is associated with relevant morbidity, which will be reviewed in the following (4.1./2.). According to the literature, complication rates of up to 83%.[14–16] Michel *et al*., report an early complication rate of 50.8% in their series of primary PCNL patients.[17]

### SURGICAL COMPLICATIONS OF PERCUTANEOUS NEPHROLITHOTOMY

Intra- and postoperative hemorrhage is one of the most frequent complications in percutaneous renal surgery. Transfusion rates of up to 34% have been described.[18–22] Delayed postoperative bleeding occurs in about 1% of all PCNL patients,[23] with the development of arteriovenous fistula or pseudoaneurysm being the most frequent cause.[24]

Lee *et al*., report radiographic proven extravasation of the collecting system in 7% of their series of 580 cases.[25] Stricture and infundibular stenosis are rare and reported in 1-2% of the patients[18,26] postoperatively.

Another major issue with PCNL is injury of adjacent structures and organs. Although mostly associated with supracostal access[27] pneumothorax and hydrothorax are reported in 4% respectively 8% of the patients.[28,29] Perforation of the bowel is described in up to 1% of the cases.[30,31]

### Medical complications of percutaneous nephrolithotomy

Sepsis, as a major complication occurs in up to 4.7% of the patients.[32,33] Septicemia can result if the stones are infected or as introduced infection via the access to the kidney. Fluid overload and absorption through extravasation or renal injury can cause serious hypertension and hypoxemia.[5] Mortality rates of approximately 0.5% are published.[34]

## RETROGRADE INTRARENAL SURGERY

Retrograde intrarenal surgery for larger (above 1 cm) lower pole calculi became possible because of the constant technical improvements in semi-rigid and flexible renoscopes, in laser technique and in ongoing improvement of working elements, such as tipless Nitinol baskets. The use of the Holium:YAG laser, after its introduction in 1994 in combination with 200 µm diameter flexible laser-fibers allow stone workup in virtually every location of the collecting system.[35] All these developments allow greater therapeutic efficacy to all aspects of the urinary tract, especially when dealing with calculi of the lower pole.[36,37] Furthermore, RIRS can be carried out safely in patients with contraindications to SWL or PCNL, like morbid obesity, severe kyphoscoliosis or renal ectopia.[38] Patients with complex co-morbid medical conditions and even patients with bleeding diathesis can be treated.

### Limitations and complications of retrograde intrarenal surgery

Inability to access lower pole calculi is rare, occurring in about 1% of the cases[8] and stone-free rates are high within one or two procedures, reaching 89% respectively 95% for lower pole calculi larger than 1 cm.[8,39]

Reported complications are minor. Postoperative colic rates are reported in 3.5-9%.[40,41] Postoperative pyelonephritis and gross hematuria,[8] occur in less than 3% of the cases.

Major complications are extremely rare. Major perforation is reported in approximately 1% of the cases.[39] The risk of postoperative stricture of the ureter is way under 1%, since the diameter of the used instruments has decreased as well as stone-fragmentation devices have improved.[37] Urinomata, urosepsis or ureteral avulsion has not been reported in recent larger series including almost 1500 procedures.[36,38,39]

### Costs of retrograde intrarenal surgery

An important issue is the cost of flexible ureteronoscopy. Maintenance cost of flexible renoscopes is known to be high. Maintenance cost of US$ 12.000 in 100 flexible renoscopies is described.[42] According to Collins, up to 70% of all damage is caused by scratching or perforation of the working channel with the sharp-edged tip of the laser fiber.

To overcome this problem, Herrmann *et al*., report their experience using the FlexGuard laser fiber insertion sheath. Hereby safe introduction of the laser fiber in a deflected renoscope becomes possible without relevant loss of performance. This device should significantly reduce the maintenance cost of flexible renoscopies.[43]

## CONCLUSION

Despite missing credit within the urological guidelines, the technical development in laser technology as well as significant improvement in flexible renoscopes had made RIRS for larger lower pole stones possible. Even more, due to these developments morbidity has decreased significantly and major complications are extremely rare. Ongoing technical developments, like the “chip-on-the-tip”-technique will add even more power to RIRS. Even if associated with a second procedure in some patients, a high stone-free rate can be achieved with one single procedure. The low complication rate gives RIRS for lower pole stones superiority over the invasive percutaneous approach, which is associated with significant morbidity, even in experienced hands.
